# Enhancing performance through biochemical monitoring and nutritional support in female weightlifters during pre-competition weight reduction: a randomized trial

**DOI:** 10.1080/15502783.2024.2435542

**Published:** 2024-11-28

**Authors:** Liang Yu, Liang Cheng

**Affiliations:** a Sichuan Sports College, Human Movement Science, Chengdu, China; b Sichuan Academy of Chinese Medicine Sciences, Chengdu, China; c Chengdu Sport University, School of Sports Medicine and Health, Chengdu, China

**Keywords:** Female weightlifters, pre-competition weight reduction, biochemical monitoring, fatigue, sports nutrition

## Abstract

**Objective:**

Weight reduction is a common practice among female weightlifters before competitions to qualify for specific weight classes. However, this process can adversely affect their physical performance and health. This study aimed to investigate the impact of physiological and biochemical monitoring and nutritional support on the competitive state of female weightlifters during the pre-competition weight reduction period.

**Methods:**

A randomized controlled trial was conducted with 28 female weightlifters from Sichuan Province, China, randomly assigned to the experimental group (*n* = 14) or the control group (*n* = 14). Both groups followed their regular training schedules. The experimental group received personalized nutritional monitoring and supplementation, including detailed assessments, dietary logs, weekly consultations, and targeted supplements based on biochemical indicators and training needs. The control group continued with their standard diet and training without additional interventions. Serum indicators, fatigue scales, and Pittsburgh Sleep Quality Index (PSQI) scores were monitored (a total of 5 tests).

**Results:**

The experimental group exhibited a significant reduction in creatine kinase levels by the fourth week compared to both baseline and the first week (*p* < 0.05), and maintained higher levels of testosterone, testosterone/cortisol ratio, and hemoglobin than the control group from the second week onwards (*p* < 0.05). Conversely, the control group showed an increase in creatine kinase and a decrease in testosterone and testosterone/cortisol ratio over the same period (*p* < 0.05). By the fourth week, the experimental group also reported lower fatigue and better sleep quality, as indicated by lower PSQI scores, compared to the control group (*p* < 0.001).

**Conclusion:**

Personalized nutritional supplementation has an improving effect on biochemical indicators, fatigue, and sleep quality in female weightlifters during the pre-competition weight reduction period. Implementing biochemical monitoring and personalized nutritional support during this period is a key strategy for enhancing the competitive performance of female weightlifters.

## 1. Introduction

Weightlifting is a sport that demands exceptional explosive power and technical skill, placing stringent requirements on athletes’ strength, speed, technique, and psychological quality [1,2]. In weightlifting competitions, athletes are also categorized based on weight classes, making pre-competition weight reduction a necessary means for many athletes to meet the minimum weight requirements [3,4]. However, the weight reduction process may be accompanied by a reduction in energy and nutrient intake, which can adversely affect the physiological functions and athletic performance of athletes [5,6]. With the advancement of sports training science, an increasing number of studies have begun to focus on the physiological impact of pre-competition weight reduction and nutritional support for athletes [7–9]. Improper weight reduction methods can lead to dehydration, electrolyte imbalances, muscle breakdown, increased fatigue, and insomnia, which can in turn affect the competitive state and health of athletes [10,11]. Therefore, developing a scientific weight reduction plan and nutritional supplementation strategy is crucial for athletes’ pre-competition preparation. Physiological and biochemical monitoring is crucial for female weightlifters as it provides insights into their energy metabolism, muscle health, and hormonal balance, which are essential for optimizing performance and health [12]. Key indicators include blood urea, creatine kinase, and hormone levels, particularly female sex hormones such as testosterone and cortisol [13]. These indicators are closely related to the effectiveness of nutritional supplementation plans, as they can reflect the body’s response to training and diet adjustments during the pre-competition weight reduction period. Additionally, specific physiological and biochemical indicators are crucial for assessing the performance of female weightlifters. For instance, the testosterone/cortisol ratio is a key indicator of the balance between anabolic and catabolic metabolism in athletes. Testosterone levels are directly related to muscle mass, strength, and recovery capabilities, while elevated cortisol levels may suppress muscle synthesis and increase catabolism [14]. Creatine kinase levels are an important biochemical marker for assessing muscle damage and recovery, with increased levels often associated with muscle injury and inflammatory responses [15]. Additionally, hemoglobin levels are directly related to an athlete’s endurance and oxygen-carrying capacity, which are particularly important for sports like weightlifting that require both explosive power and endurance.

Despite existing research providing some guiding principles on pre-competition weight reduction and nutritional management for weightlifters, these studies use uniform standards to guide all weightlifters, neglecting individual differences among athletes and specific project requirements [16–18]. In addition, existing research has mainly focused on the short-term effects after weight reduction [19,20], with relatively less research on continuous physiological monitoring [21] and nutritional support during the weight reduction period [22]. There are significant individual differences in the physiological needs and nutritional responses of weightlifters during the pre-competition weight reduction period, and a one-size-fits-all nutritional supplementation plan is difficult to meet the needs of all athletes. A personalized nutritional supplementation plan can be adjusted according to athletes’ biochemical indicators, body composition, training load, and other factors, which is more conducive to optimizing athletes’ physiological state and athletic performance [23].

This study, based on existing research and with a study period of one month before the 2024 Sichuan Provincial Weightlifting Team competition, proposes a personalized nutritional supplementation plan based on physiological and biochemical monitoring, and verifies it using the method of randomized controlled trials. During the pre-competition weight reduction, significant changes in female sex hormones occur, which can affect muscle mass, strength, and recovery [24]. Monitoring these hormonal fluctuations is essential for adjusting nutritional strategies to maintain physiological balance and support the athletes’ competitive performance [25]. The purpose is to explore the application effect of personalized physiological and biochemical monitoring and nutritional supplementation plans during the pre-competition weight reduction period of weightlifters, and to evaluate their impact on athletes’ physiological state, fatigue, and sleep. It aims to provide a scientific basis for the pre-competition training and nutritional management of weightlifters, optimize athletes’ weight reduction strategies, and improve athletes’ competitive performance.

The research hypothesis is that a personalized nutritional supplementation plan based on physiological and biochemical monitoring will help maintain the physiological balance of female weightlifters during the pre-competition weight reduction period and reduce fatigue and sleep disorders.

## 2. Materials and methods

### 2.1. Participants

This study was approved by the Ethics Committee of Sichuan Sports College for human experimentation (SSC20246). This study is registered with ClinicalTrials. gov (NCT06553963). The athletes in the study came from the Sichuan Provincial Weightlifting Project Management Center in China, ensuring the consistency of the competitive level and training conditions of the samples. Through communication with coaches and self-reporting by athletes, athletes who met the conditions were preliminarily screened. A professional medical team conducted a comprehensive physical examination of the pre-screened athletes to exclude individuals with health risks.

Considering the limited number of female weightlifters in Sichuan Province, China, this study included all available athletes as participants, resulting in a relatively small sample size. This is similar to previous studies with small samples, all of which were specifically focused on weight loss among elite athletes [24,26,27].

Inclusion Criteria: female weightlifters aged 18 to 30; at least 5 years of professional training experience; currently undergoing systematic training at the Sichuan Provincial Weightlifting Project Management Center; passed a comprehensive health examination by a professional medical team, with no preexisting health risks affecting study outcomes; signed an informed consent form in line with the ethical standards of the Declaration of Helsinki.

Exclusion Criteria: history or presence of significant lower limb joint injuries that could worsen due to training or weight reduction; use of prescription medications affecting mood and sleep, potentially confounding study outcomes related to fatigue and sleep quality; abnormal cardiovascular function, as determined by medical examination, posing a risk during intense training and weight reduction periods.

**Figure 1.**
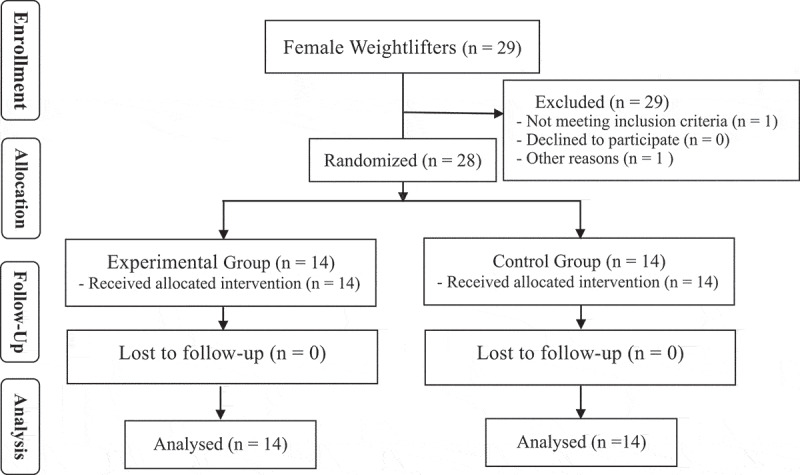
Experimental flowchart.

This study adhered to the design principles of a randomized controlled trial and conducted a strict randomization grouping process for participants. Initially, all eligible female weightlifters were numbered through a computer-generated random number sequence to ensure the randomness of the grouping. Subsequently, a simple random sampling method was used to equally allocate athletes to the experimental group (*n* = 14) and the control group (*n* = 14) (Figure 1). According to the weight divisions set by the International Weightlifting Federation for the senior women’s category, the experimental group had one participant at 48 kg, one at 53 kg, four at 58 kg, four at 63 kg, and four at 69 kg; the control group had two participants at 48 kg, two at 58 kg, six at 63 kg, and four at 69 kg. This process was carried out by an independent researcher not involved in subsequent research operations to ensure the fairness and double-blind nature of the grouping. The baseline data between groups (Table 1) showed no statistically significant differences (*p* > 0.05).

**Table 1.** Participant baseline information.

Indicator	Experimental group	Control group	*p*
n	14	14	
Age (years)	20.4 ± 2.8	20.9 ± 2.2	0.397
Height (cm)	162.5 ± 4.6	162.2 ± 5.1	0.868
Weight (kg)	62.7 ± 6.1	63.0 ± 6.3	0.950
Training years (years)	8.2 ± 1.5	8.5 ± 1.6	0.681
Blood urea (mmol/L)	5.3 ± 1.1	5.2 ± 1.2	0.820
Creatine kinase (U/L)	290.6 ± 45.2	283.9 ± 40.7	0.688
Serum testosterone (nmol/L)	1.82 ± 0.31	1.81 ± 0.29	0.948
Serum cortisol (nmol/L)	138.3 ± 32.6	140.5 ± 34.8	0.864
Testosterone/cortisol ratio	0.014 ± 0.003	0.014 ± 0.004	0.931
Hemoglobin (g/dL)	14.5 ± 0.8	14.6 ± 0.9	0.764
Fatigue score (points)	6.2 ± 1.5	6.1 ± 1.7	0.943
Pittsburgh Sleep Quality Index scores	5.9 ± 1.8	6.0 ± 2.0	0.627

### 2.2. Procedures

This study took place from March to April 2024, spanning a total of 4 weeks. Initially, All participants followed a standardized training plan set by their coaches during the study period. The plan included training 6 days a week, with each training session lasting 2–3 hours, covering strength training, technical drills, and aerobic endurance training. Specifically, strength training included variations of squats, clean and jerk, and snatch, technical drills focused on perfecting weightlifting techniques and details, and aerobic endurance training was enhanced through running and cycling. Furthermore, each athlete’s training plan was fine-tuned based on their individual physical fitness and competitive state to ensure the safety and effectiveness of the training.

On this foundation, participants in the experimental group received a four-week personalized nutritional monitoring and supplementation program. This program was tailored by sports nutrition experts according to each athlete’s biochemical indicators, body weight, training load, and personal dietary habits. It specifically included, but was not limited to: Nutritional Assessment, where a detailed nutritional evaluation was conducted for each athlete before the start of the study, including dietary history, biochemical indicator analysis, etc.; Dietary Logs, where athletes were asked to record their daily food intake to allow the nutrition team to monitor and adjust in real time; Nutritional Consultations: Weekly face-to-face consultations with nutrition experts to discuss dietary plans and resolve any questions or issues; Additionally, this study monitored the levels of sex hormones, including testosterone and cortisol, among participants to assess their impact on training adaptation and competitive status. The nutritional supplementation plan for the experimental group took into account the fluctuations in sex hormone levels to ensure the physiological balance of the athletes during the weight reduction period.

Prevalence of Sleep Disorders: Two athletes (14.3%) in the experimental group reported sleep disorders, while five (35.7%) in the control group did so. Creatine Kinase Levels: The average creatine kinase levels in the experimental group decreased from 290.6 ± 45.2 U/L at baseline to 260.0 ± 35.6 U/L by the fourth week, while the control group increased from 283.9 ± 40.7 U/L at baseline to 340.0 ± 50.8 U/L by the fourth week. Testosterone Levels: The average testosterone levels in the experimental group remained stable from 1.82 ± 0.31 nmol/L at baseline to 1.79 ± 0.27 nmol/L by the fourth week, while the control group decreased from 1.81 ± 0.29 nmol/L at baseline to 1.50 ± 0.25 nmol/L by the fourth week.

### 2.3. Nutritional supplements

All nutritional supplements used in the study met international anti-doping standards, ensuring the safety and legality of the research. Compliance with the nutritional supplementation plan was assessed through regular checks and interviews, with no participant reporting any serious adverse events or health issues.

Adjusting the types and dosages of nutritional supplements based on weekly biochemical monitoring results, aimed at optimizing athletes’ biochemical indicators, such as reducing blood urea and cortisol, and increasing hemoglobin (Nutritional supplement name: Zinc Magnesium Multi-Vitamin Sports Nutrition Powder, 180 g/bag; Origin: Beijing, China, Winpowerdata Technology (Beijing) Co., Ltd.; Ingredients: Rice bran lipid alcohol, magnesium, zinc, vitamin C, taurine, vitamin B1, vitamin B2, vitamin B6; Dosage: 1–2 bags daily). Zinc and magnesium were chosen as ingredients in the supplement due to their critical roles in supporting athletes’ immune systems, protein synthesis, and energy metabolism; Reducing creatine kinase (Nutritional supplement name: Endurance Sugar Pump, 70 g/bag; Origin: Beijing, China, Weite Beijing Co., Ltd.; Ingredients: Soluble barley starch 62 g, fructose 1,6-diphosphate 6 g, electrolytes; Dosage: 1–2 bags during daily training); Enhancing testosterone (Nutritional supplement name: Testosterone Synthesis Pump, 1 capsule/2 grams; Origin: Beijing, China, Weite Beijing Co., Ltd.; Ingredients: Super testosterone complex, testosterone action amplification factor, testosterone receptor sensitizer, 5-level male testosterone optimization factor, aspartic acid, Rhodiola; Dosage: 6 capsules before daily training); Promoting sleep (Nutritional supplement name: Melatonin Tablets, 1 tablet/0.5 grams; Origin: Beijing, China, Dongli Beijing Co., Ltd.; Ingredients: Vitamin B6, 1600 mg/100 g, melatonin, 500 mg/100 g; Dosage: 1 tablet before bedtime); Control group participants continued to follow their regular diet and training plan without additional nutritional intervention.

### 2.4. Monitoring indicators and methods

In this study, we also monitored the menstrual cycles of the participants and documented potential impacts. To control the effects of the menstrual cycle on training and competitive status, participants were advised to follow specific dietary and supplementation strategies while avoiding medications that could affect hormone levels. This study conducted five tests on all participants, at baseline (before weight reduction), week one, week two, week three, and week four (1–2 days before the competition). The time was fixed on Sunday (6:30–7:30 AM). The Pittsburgh Sleep Quality Index (PSQI) test was conducted twice (at baseline and week four).

#### 2.4.1. Serum testing

Approximately 2 ml of fasting venous blood was taken from the participants’ elbow. For each indicator, specific procedures were followed: Blood urea and creatine kinase were measured using the Beckman Coulter Automated Biochemical Analyzer, with samples collected in the fasting state for accuracy. Testosterone and cortisol levels were assessed using the Beckman Coulter Chemiluminescence Immunoassay Analyzer, which provides sensitive detection of these hormones. Hemoglobin was measured using the HemoCue Hb 301 hemoglobinometer, which offers rapid and reliable results.

#### 2.4.2. Scale testing

Participants’ fatigue scores were tested, with participants asked to mark their fatigue score on a 10 cm line, with the far left indicating no fatigue and the far right indicating severe fatigue [28,29]. The Pittsburgh Sleep Quality Index (PSQI) is a questionnaire used to assess the sleep quality of the subject over the past month, composed of 18 self-rated items forming 7 components, with a total score ranging from 0 to 21 points, the higher the score, the worse the sleep quality. The scale’s reliability for Chinese subjects is between 0.65 and 0.84, and the validity is greater than 0.85 [30,31].

### 2.5. Statistical analysis

SPSS 20.0 was used to process all measured data as mean ± standard deviation. This is a 2 (group) × 5 (time) experimental design. The Shapiro-Wilk test was used to measure the normality of the data and the homogeneity of variance. If the data did not conform to a normal distribution or homogeneity of variance, the Kruskal-Wallis test was used to compare differences at different time points. If the data conformed to a normal distribution and homogeneity of variance, a two-way analysis of variance was used to analyze the main effects of group and time, as well as whether there was an interaction between group and time. If there was an interaction, analyze whether there is a separate effect of time or group; if there was no interaction, analyze whether there is a main effect of group or time [32,33]. Post-hoc comparisons within groups at different time points were made using LSD, ensuring that the overall Type I error rate for each analysis of variance was no greater than 0.05. The level of significance α = 0.05.

## 3. Results

**Figure 2.**
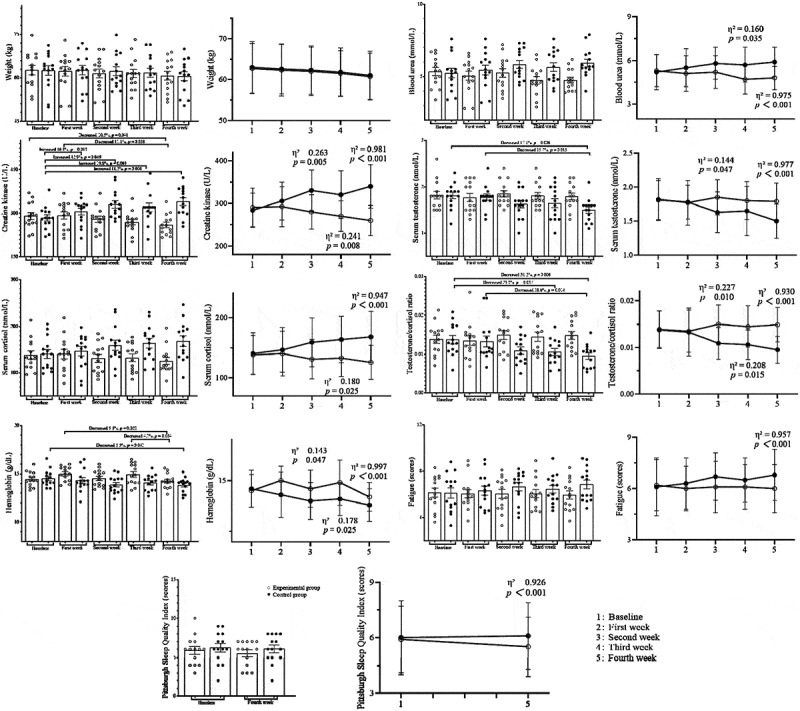
Comparisons within groups at different time points and between groups at the same time points.

Comparisons within groups at different time points and between groups at the same time points are shown in Figure 2. The Shapiro-Wilk test indicated that the data met the criteria for normal distribution and homogeneity of variance. Consequently, two-way analysis revealed significant interactions between group and time for blood urea (*p* = 0.023, F = 5.515, η^2^ = 0.096), creatine kinase (*p* < 0.001, F = 14.081, η^2^ = 0.213), cortisol (*p* = 0.036, F = 4.643, η^2^ = 0.082), and the testosterone/cortisol ratio (*p* = 0.013, F = 6.624, η^2^ = 0.113), prompting further investigation into the separate effects of time or group. Other indicators did not show an interaction between group and time, leading to further assessment of the main effects of group or time. Post-hoc comparisons were conducted using the LSD method.

### 3.1 Within-Group Comparisons

For the experimental group, weight, blood urea, testosterone, cortisol, testosterone/cortisol ratio, and fatigue scores at different time points showed no statistically significant differences (*p* > 0.05), and the PSQI scores at week four compared to baseline were also not significantly different (*p* > 0.05). Creatine kinase decreased by 10.5% and 11.1% at the fourth week compared to baseline and first week, respectively (95% CI: −62.192 ~ 0.077, *p* = 0.041; 95% CI: −64.092 ~ −1.823, *p* = 0.038). Hemoglobin decreased by 5.3% and 4.7% at the fourth week compared to first and third weeks, respectively (95% CI: −8.456 ~ 0.431, *p* = 0.022; 95% CI: −1.331 ~ 0.054, *p* = 0.034). For the control group, weight, blood urea, cortisol, and fatigue scores at different time points showed no statistically significant differences (*p* > 0.05), and the PSQI scores at fourth week compared to baseline were not significantly different (*p* > 0.05). Creatine kinase increased by 16.4%, 12.9%, and 19.8% at first, second and third weeks, respectively, compared to baseline (95% CI: −14.622 ~ 58.050, *p* = 0.013; 95% CI: 10.264 ~ 82.936, *p* = 0.049; 95% CI: 0.178 ~ 72.850, *p* = 0.003). Compared to first week, it increased by 11.2% at fourth week (95% CI: −1.943 ~ 70.729, *p* = 0.006). Testosterone decreased by 17.1% and 15.7% at fourth week compared to baseline and firs week, respectively (95% CI: −0.533 ~ −0.095, *p* = 0.006; 95% CI: −0.497 ~ −0.060, *p* = 0.013). The testosterone/cortisol ratio decreased by 23.2% and 31.2% third and fourth weeks, respectively, compared to baseline (95% CI: 0.0002 ~ 0.0061, *p* = 0.037; 95% CI: 0.0013 ~ 0.0072, *p* = 0.006), and decreased by 28.6% at fourth week compared to first week (95% CI: 0.0008 ~ 0.0067, *p* = 0.014). Hemoglobin decreased by 5.5% at fourth week compared to baseline (95% CI: −1.389 ~ 0.025, *p* = 0.042).

### 3.2 Between-Group Comparisons

At the first week, no statistically significant differences were found between groups for any indicators (*p* > 0.05). At the second week, the experimental group had lower creatine kinase (η^2^ = 0.263, *p* = 0.005) and higher testosterone (η^2^ = 0.144, *p* = 0.047), testosterone/cortisol ratio (η^2^ = 0.227, *p* = 0.010), and hemoglobin (η^2^ = 0.143, *p* = 0.047) than the control group. At the third week, blood urea (η^2^ = 0.160, *p* = 0.035), creatine kinase (η^2^ = 0.241, *p* = 0.008), and cortisol (η^2^ = 0.180, *p* = 0.025) were lower, while the testosterone/cortisol ratio (η^2^ = 0.208, *p* = 0.015) and hemoglobin (η^2^ = 0.178, *p* = 0.025) were higher in the experimental group. At the fourth week, blood urea (η^2^ = 0.975), creatine kinase (η^2^ = 0.981), cortisol (η^2^ = 0.947), fatigue scores (η^2^ = 0.957), and PSQI scores (η^2^ = 0.926) were lower, and testosterone (η^2^ = 0.977), testosterone/cortisol ratio (η^2^ = 0.930), and hemoglobin (η^2^ = 0.997) were higher in the experimental group compared to the control group (*p* < 0.001).

## 4. Discussion

The purpose of this study was to explore the effects of a personalized physiological and biochemical monitoring and nutritional supplementation program during the pre-competition weight reduction period for weightlifters, assessing its impact on athletes’ physiological status, fatigue, and sleep. This study confirmed our research hypothesis that a personalized nutritional supplementation program based on physiological and biochemical monitoring would help maintain physiological balance, reduce fatigue, and alleviate sleep disturbances in female weightlifters during the pre-competition weight reduction period.

This study demonstrated that, compared to the control group, the experimental group had a significant reduction in blood urea (weeks three to four) and creatine kinase (weeks two to four) levels during the weight reduction period, supporting our hypothesis that personalized nutritional supplementation can effectively mitigate muscle damage and protein breakdown caused by weight reduction. The improvements in blood urea [34] and creatine kinase [35] reflect the effectiveness of the nutritional program in maintaining muscle health and reducing metabolic stress. Furthermore, the relatively stable testosterone levels and a slight increase in cortisol levels, along with an improved testosterone/cortisol ratio in the experimental group, further indicate that personalized nutritional supplementation helps maintain the balance between anabolic and catabolic metabolism [36]. This balance is crucial for reducing athletes’ fatigue and promoting recovery after training [37], which aligns with our research hypothesis.

Most current research introduces weightlifting strategies for weightlifters [1–3,15,19] or general nutritional advice [38–40]. Few studies have explored the application effects of personalized physiological and biochemical monitoring and nutritional supplementation programs during the pre-competition weight reduction period for weightlifters. This study provided a customized nutritional plan for each athlete through meticulous physiological and biochemical monitoring. The reasons for this are as follows: First, the personalized nutritional supplementation program helped athletes maintain energy and metabolic balance during weight reduction by accurately adjusting the intake of macronutrients and micronutrients. This may have reduced muscle breakdown and kept the levels of blood urea and creatine kinase stable; second, the regulatory effect of nutritional supplementation on hormone levels helped maintain testosterone levels during weight reduction, supporting anabolic processes, while proper cortisol management may have inhibited excessive catabolism [41], which was reflected in the changes in the testosterone/cortisol ratio; finally, adequate nutritional support, especially sufficient protein and antioxidant intake, accelerated the recovery process after muscle damage, reducing the release of creatine kinase, thereby maintaining athletes’ muscle function and athletic ability.

It is worth noting that fatigue and sleep quality in the experimental group also improved to some extent by the fourth week. This supports the research hypothesis. The nutritional supplementation program, by maintaining or improving biochemical indicators such as hemoglobin and creatine kinase levels, helped reduce muscle fatigue and speed up recovery [42]. By regulating energy metabolism, it increased the athletes’ energy levels and endurance, thereby reducing post-training fatigue. In addition, antioxidants and other micronutrients included in the nutritional supplementation can reduce oxidative stress and muscle damage [43], further lowering fatigue levels. The improvement in sleep quality may be related to sleep-promoting nutrients (tryptophan and magnesium) contained in the personalized nutritional supplementation program. These nutrients help regulate the sleep-wake cycle and improve deep sleep and overall sleep quality [44]. They may indirectly improve sleep by reducing fatigue and stress. The improvement in sleep quality is crucial for athletes’ recovery, immune function, and mental health [45]. Studies suggest a close interrelationship between athletes’ fatigue and sleep quality [46]. The improvements observed in the experimental group in terms of biochemical indicators, fatigue levels, and sleep quality may be attributed to the effects of specific nutrients in the supplements. For instance, magnesium and zinc in the supplements help regulate nervous system function and reduce oxidative stress, thereby reducing muscle fatigue [47]. Magnesium, acting as a natural muscle relaxant, helps reduce muscle tension and soreness post-exercise, while zinc is essential for cell repair and immune function, aiding in the recovery process [48]. Additionally, B vitamins and melatonin in the supplements help regulate the sleep-wake cycle and improve sleep quality. B vitamins are crucial for energy metabolism and nervous system function, while melatonin, a hormone that regulates the sleep-wake cycle, helps improve deep sleep and overall sleep quality [49]. Persistent fatigue may disrupt normal sleep patterns, leading to decreased sleep quality. Conversely, improved sleep quality helps reduce fatigue, forming a virtuous cycle. The high compliance of the study participants with the personalized nutritional supplementation program indicates that the plan can be accepted and implemented by athletes. No serious adverse events or health issues were observed during the study, confirming the safety of the program.

In light of this study’s findings, we advise practitioners to employ biochemical monitoring and tailored nutritional support for female weightlifters during the pre-competition weight reduction phase. Regular assessments of blood markers like creatine kinase, testosterone, cortisol, and hemoglobin should guide nutritional adjustments. Personalized supplements should also be provided to maintain energy balance, reduce muscle damage, and enhance sleep quality, ultimately improving athletes’ performance and health.

This study acknowledges several limitations that will guide the direction of future research. Firstly, the sample size, though inclusive of all eligible female weightlifters in Sichuan Province, was relatively small, the limited sample size of this study may restrict statistical power, particularly in detecting smaller effect sizes. A smaller sample size can lead to insufficient power in statistical tests, preventing the detection of all relevant differences. Secondly, the study’s duration was confined to a short period, precluding the assessment of long-term outcomes associated with the personalized nutritional supplementation program. Additionally, the scope of this study did not encompass a mental health questionnaire or comprehensive assessments of metabolism and body composition, aspects that are critical for a holistic evaluation of the athletes’ health and performance. These omissions will be rectified in subsequent studies, which will not only expand the sample size and extend the study period but also delve into the long-term impacts of various nutritional supplementation strategies on the health and athletic performance of weightlifters.

## 5. Conclusion

Personalized nutritional supplementation has an improving effect on biochemical indicators, fatigue, and sleep quality in female weightlifters during the pre-competition weight reduction period. Implementing biochemical monitoring and personalized nutritional supplementation during this period is a key strategy for enhancing the competitive performance of female weightlifters.

## Data Availability

The raw data supporting the conclusions of this article will be made available by the authors, without undue reservation.

## References

[1] Nelson M, Jette S. Muscle moves mass: deconstructing the culture of weight loss in American Olympic weightlifting. Int Rev Sociol Sport. 2023;58(5):765–15. doi: 10.1177/10126902221120183

[2] Crenshaw K, Zeppieri G, Hung CJ, et al. Olympic weightlifting training for sprint performance in athletes: a systematic review with meta-analysis. Int J Sports Med. 2024;45(6):411–421. doi: 10.1055/a-2161-4867 37640059 PMC11153037

[3] Nolan D, Lynch AE, Self-Reported Prevalence EB. Magnitude, and methods of rapid weight loss in male and female competitive powerlifters. J Strength Cond Res. 2022;36(2):405–410. doi: 10.1519/JSC.0000000000003488 31904717

[4] Kwan K, Prevalence HE. Magnitude, and methods of weight cutting used by world class powerlifters. J Strength Cond Res. 2022;36(4):998–1002. doi: 10.1519/JSC.0000000000004199 35319002

[5] Evans C, Stull C, Sanders G, et al. Weight cutting in female UFC fighters. J Int Soc Sports Nutr. 2023;20(1):2247384. doi: 10.1080/15502783.2023.2247384 37621001 PMC10453969

[6] Sung JY, Lee JH, Lee KL. Analysis of the diet, weight-loss behavior, and nutritional knowledge of athletes and coaches in weightclass sports: influence of a coach’s nutritional knowledge on athletes. J Int Soc Sports Nutr. 2024;21(1):2405159. doi: 10.1080/15502783.2024.2405159 39287144 PMC11409413

[7] Štangar M, Štangar A, Shtyrba V, et al. Rapid weight loss among elite-level judo athletes: methods and nutrition in relation to competition performance. J Int Soc Sports Nutr. 2022;19(1):380–396. doi: 10.1080/15502783.2022.2099231 35859622 PMC9291696

[8] Burke LM, Slater GJ, Matthews JJ, et al. ACSM expert consensus statement on weight loss in weight-category sports. Curr Sports Med Rep. 2021;20(4):199–217. doi: 10.1249/JSR.0000000000000831 33790193

[9] Lakicevic N, Paoli A, Roklicer R, et al. Effects of rapid weight loss on kidney function in combat sport athletes. Medicina (B Aires). 2021;57(6):551. doi: 10.3390/medicina57060551 PMC8229569 34072641

[10] Roklicer R, Rossi C, Bianco A, et al. Prevalence of rapid weight loss in Olympic style wrestlers. J Int Soc Sports Nutr. 2022;19(1):593–602. doi: 10.1080/15502783.2022.2119095 36250149 PMC9559051

[11] Papadopoulou SK, Dalatsi VA, Methenitis SK, et al. Nutritional routine of tae kwon do athletes prior to competition: what is the impact of weight control practices? J Am Coll Nutr. 2017;36(6):448–454. doi: 10.1080/07315724.2017.1319305 28628394

[12] Lee EC, Fragala MS, Kavouras SA, et al. Biomarkers in sports and exercise: tracking health, performance, and recovery in athletes. J Strength Cond Res. 2017;31(10):2920–2937. doi: 10.1519/JSC.0000000000002122 28737585 PMC5640004

[13] Sims ST, Kerksick CM, Smith RAE, et al. International society of sports nutrition position stand: nutritional concerns of the female athlete. J Int Soc Sports Nutr. 2023;20(1):2204066. doi: 10.1080/15502783.2023.2204066 37221858 PMC10210857

[14] Kraemer WJ, Ratamess NA, Hymer WC, et al. Growth Hormone(s), testosterone, insulin-like growth factors, and cortisol: roles and integration for cellular development and growth with exercise. Front Endocrinol (Lausanne). 2020;11:33. doi: 10.3389/fendo.2020.00033 32158429 PMC7052063

[15] Markus I, Constantini K, Hoffman JR, et al. Exercise-induced muscle damage: mechanism, assessment and nutritional factors to accelerate recovery. Eur J Appl Physiol. 2021;121(4):969–992. doi: 10.1007/s00421-020-04566-4 33420603

[16] Durguerian A, Bougard C, Drogou C, et al. Weight loss, performance and psychological related states in high-level weightlifters. Int J Sports Med. 2016;37(3):230–238. doi: 10.1055/s-0035-1555852 26701827

[17] Gee TI, Campbell P, Bargh MJ, et al. Rapid weight loss practices within Olympic weightlifters. J Strength Cond Res. 2023;37(10):2046–2051. doi: 10.1519/JSC.0000000000004507 37729517

[18] Andersen RE, Barlett SJ, Morgan GD, et al. Weight loss, psychological, and nutritional patterns in competitive male body builders. Int J Eat Disord. 1995 Jul;18(1):49–57. doi: 10.1002/1098-108X(199507)18:1<49::AID-EAT2260180106>3.0.CO;2-C 7670443

[19] Travis SK, Mizuguchi S, Stone MH, et al. Preparing for a National weightlifting championship: a case series. J Strength Cond Res. 2020;34(7):1842–1850. doi: 10.1519/JSC.0000000000003312 31373973

[20] Campbell P, Martin D, Bargh MJ, et al. A comparison of rapid weight loss practices within international, national and regional powerlifters. Nutr Health. 2023:2601060231201892. doi: 10.1177/02601060231201892 37697737 PMC12174615

[21] Aronne LJ, Hall KD, Jakicic J M, et al. Describing the weight-reduced state: physiology, behavior, and interventions. Obesity. 2021;1 Suppl 29(Suppl 1):S9–S24. doi: 10.1002/oby.23086 Obesity (Silver Spring). 33759395 PMC9022199

[22] Manore MM. Weight management for athletes and active individuals: a brief review. Sports Med. 2015; 45 (Suppl 1):S83–92. doi: 10.1007/s40279-015-0401-0 26553496 PMC4672016

[23] Amawi A, AlKasasbeh W, Jaradat M, et al. Athletes’ nutritional demands: a narrative review of nutritional requirements. Front Nutr. 2024;10:1331854. doi: 10.3389/fnut.2023.1331854 38328685 PMC10848936

[24] Yu L, Lei L, Cheng L. Influence of slow and rapid weight loss periods on physiological performance, mood state and sleep quality in male freestyle wrestlers: a study from Sichuan Province, China. Front Psychol. 2024;15:1445810. doi: 10.3389/fpsyg.2024.1445810 39474087 PMC11518747

[25] Pedlar CR, Newell J, Lewis NA. Blood biomarker profiling and monitoring for high-performance physiology and nutrition: current perspectives, limitations and recommendations. Sports Med. 2019;49(Suppl 2):185–198. doi: 10.1007/s40279-019-01158-x PMC6901403 31691931

[26] Reljic D, Jost J, Dickau K, et al. Effects of pre-competitional rapid weight loss on nutrition, vitamin status and oxidative stress in elite boxers. J Sports Sci. 2015;33(5):437–448. doi: 10.1080/02640414.2014.949825 25259507

[27] Matthews JJ, Nicholas C. Extreme rapid weight loss and rapid weight gain observed in UK mixed martial arts athletes preparing for competition. Int J Sport Nutr Exerc Metab. 2017;27(2):122–129. doi: 10.1123/ijsnem.2016-0174 27710145

[28] Cheng L, Wang K, He B, et al. Effect of vertical vibration stimulation at different frequencies on delayed muscle soreness in athletes: a randomized trial. Front Public Health. 2022;10:980454. doi: 10.3389/fpubh.2022.980454 36311634 PMC9614366

[29] Cheng LJ, Jiang, Y Y. Effect of extracorporeal shock wave therapy on pain and forearm rotating muscle strength in patients with tennis elbow. Med Sport. 2020;73(4):661–672. doi: 10.23736/S0025-7826.20.03685-6

[30] Cheng L, Chang S, Wang B, et al. Cross-sectional study of depression tendency and sleep quality in 1352 people practicing Tai Chi. Res Sports Med. 2023;31(5):650–662. doi: 10.1080/15438627.2021.2024832 34994259

[31] Chang S, Cheng L, Liu H. Effects of three-duration Tai-Chi exercises on depression and sleep quality in older women. Eur Geriatr Med. 2024;15(4):1141–1148. doi: 10.1007/s41999-024-00981-4 38693298

[32] Cheng L, Wang K, Chang S, et al. Effects of platelet-rich plasma combined with isometric quadriceps contraction on cartilage in a rat model of knee osteoarthritis. Regen Ther. 2024;26:469–477. doi: 10.1016/j.reth.2024.06021 39070125 PMC11283084

[33] Cheng L, Chang S, He B, et al. Effects of Tai Chi and brisk walking on the bone mineral density of perimenopausal women: a randomized controlled trial. Front Public Health. 2022;10:948890. 948890. doi: 10.3389/fpubh.2022 36072375 PMC9441636

[34] Stankevych L, Zemtsova I, Khmelnytska Y, et al. Correction of endurance training and competitive activities of athletes by determining the blood urea content. Sport Mont. 2021;19(S2):131–135. doi: 10.26773/smj.210922

[35] Ovchinnikov AN, Paoli A, Seleznev , VV, et al. Measurement of lipid peroxidation products and creatine kinase in blood plasma and saliva of athletes at rest and following exercise. J Clin Med. 2022;11(11):3098. doi: 10.3390/jcm11113098 35683484 PMC9181342

[36] Ficarra G, Caccamo D, Rottura M, et al. Testosterone: cortisol ratio as a predictor of podium in adolescent rowing athletes. Heliyon. 2023;9(11):e22315. doi: 10.1016/j.heliyon.2023.e22315 38053894 PMC10694314

[37] Hayes LD, Grace FM, Baker JS, et al. Exercise-induced responses in salivary testosterone, cortisol, and their ratios in men: a meta-analysis. Sports Med. 2015;45(5):713–726. doi: 10.1007/s40279-015-0306-y 25655373

[38] Greene DA, Varley BJ, Hartwig TB, et al. A low-carbohydrate ketogenic diet reduces body Mass without compromising performance in powerlifting and Olympic weightlifting athletes. J Strength Cond Res. 2018;32(12):3373–3382. doi: 10.1519/JSC.0000000000002904 30335720

[39] Hasan MF, Bahri S, Adnyana IK. Identification of nutritional status and body composition in weightlifting athlete. J Phy Edu Sport. 2021;21(4):2308–2312. doi: 10.7752/jpes.2021.s4294

[40] King A, Kwan K, Jukic I, et al. The general nutrition practices of competitive powerlifters vary by competitive calibre and sex, weight, and age class. Eur J Nutr. 2023;62(8):3297–3310. doi: 10.1007/s00394-023-03233-6 37584786 PMC10611852

[41] Crewther BT, Cook C, Cardinale M, et al. Two emerging concepts for elite athletes: the short-term effects of testosterone and cortisol on the neuromuscular system and the dose-response training role of these endogenous hormones. Sports Med. 2011;41(2):103–123. doi: 10.2165/11539170-000000000-00000 21244104

[42] Andersson HM, Raastad T, Nilsson J, et al. Neuromuscular fatigue and recovery in elite female soccer: effects of active recovery. Med Sci Sports Exerc. 2008;40(2):372–380. doi: 10.1249/mss.0b013e31815b8497 18202563

[43] Clemente SVJ, Bustamante SÁ, Mielgo AJ, et al. Antioxidants and sports performance. Nutrients. 2023;15(10):2371. doi: 10.3390/nu15102371 37242253 PMC10220679

[44] Briskey D, Erickson J, Smith C, et al. Wild Nutrition’s food-Grown® magnesium supplementation increases sleep quality and sleep duration and reduces stress in a healthy adult population: a double-blind, randomised, placebo-controlled study. Food And Nutr Sci. 2024;15(7):509–523. doi: 10.4236/fns.2024.157034

[45] Charest J, Grandner MA. Sleep and athletic performance: impacts on physical performance, mental performance, injury risk and recovery, and mental health: an update. Sleep Med Clin. 2022;17(2):263–282. doi: 10.1016/j.jsmc.2022.03.006 35659079

[46] Aloulou A, Duforez F, Léger D, et al. The relationships between training load, type of sport, and sleep among high-level adolescent athletes. Int J Sports Physiol Perform. 2021;16(6):890–899. doi: 10.1123/ijspp.2020-0463 33631716

[47] Tardy AL, Pouteau E, Marquez D, et al. Vitamins and minerals for energy, fatigue and cognition: a narrative review of the biochemical and clinical evidence. Nutrients. 2020;12(1):228. doi: 10.3390/nu12010228 31963141 PMC7019700

[48] Souza ACR, Vasconcelos AR, Dias DD, et al. The integral role of magnesium in muscle integrity and aging: a comprehensive review. Nutrients. 2023;15(24):5127. doi: 10.3390/nu15245127 38140385 PMC10745813

[49] De Simone M, De Feo R, Choucha A, et al. Enhancing sleep quality: assessing the efficacy of a fixed combination of Linden, Hawthorn, vitamin B1, and Melatonin. Med Sci (Basel). 2023;12(1):2. doi: 10.3390/medsci12010002 38249078 PMC10801487

